# Large scale screening of commonly used Iranian traditional medicinal plants against urease activity

**DOI:** 10.1186/2008-2231-20-72

**Published:** 2012-10-31

**Authors:** Farzaneh Nabati, Faraz Mojab, Mehran Habibi-Rezaei, Kowsar Bagherzadeh, Massoud Amanlou, Behnam Yousefi

**Affiliations:** 1 Department of Medicinal Chemistry, Faculty of Pharmacy, Tehran University of Medical Sciences, Tehran, Iran; 2 Department of Pharmacognosy, School of Pharmacy, Shahid Beheshti University of Medical Sciences, Tehran, Iran; 3 School of Biology, College of Science, University of Tehran, Tehran, Iran; 4 Medicinal Plants Research Center, Tehran University of Medical Sciences, Tehran, Iran; 5 School of Advanced Medical Technologies, Tehran University of Medical Sciences, Tehran, Iran

**Keywords:** Urease inhibitor, Iranian traditional medicinal plants, *Sambucus ebulus*, *Rheum ribes*, Screening of natural products

## Abstract

**Background and purpose of the study:**

*H. pylori* infection is an important etiologic impetus usually leading to gastric disease and urease enzyme is the most crucial role is to protect the bacteria in the acidic environment of the stomach. Then urease inhibitors would increase sensitivity of the bacteria in acidic medium.

**Methods:**

137 Iranian traditional medicinal plants were examined against Jack bean urease activity by Berthelot reaction. Each herb was extracted using 50% aqueous methanol. The more effective extracts were further tested and their IC_50 _values were determined.

**Results:**

37 plants out of the 137 crude extracts revealed strong urease inhibitory activity (more than 70% inhibition against urease activity at 10 mg/ml concentration). Nine of the whole studied plants crude extracts were found as the most effective with IC_50 _values less than 500 μg/ml including; *Rheum ribes, Sambucus ebulus, Pistachia lentiscus, Myrtus communis, Areca catechu, Citrus aurantifolia, Myristica fragrans, Cinnamomum zeylanicum* and *Nicotiana tabacum.*

**Conclusions:**

The most potent urease inhibitory was observed for *Sambucus ebulus* and *Rheum ribes* extracts with IC_50 _values of 57 and 92 μg/ml, respectively.

## Introduction

Ureases (urea amidohydrolases, EC (3.5.1.5) are a group of widespread enzymes in nature, classified as the most proficient enzymes (with proficiency more than 10^14^), stand as protagonist in biochemistry for several reasons. Urease was the first ureolytic enzyme obtained and named in the late nineteenth century, with landmark significance in enzymology as the first enzyme crystallized (in 1926 by Sumner) to approve the proteinous nature of the enzymes [1]. Also, as ascertained by Dixon et al. in 1975, urease was the first enzyme shown to possess nickel ions in its active site, essential for activity [2]. Since its substrate; urea is pervasively available in nature, urease was important to provide organisms with nitrogen in the form of ammonia for growth [3]. Despite the diversity in the molecular structures of urease, the amino acid sequences of the active sites are principally similar in all of the known them and consequence of this fact is the same catalytic mechanism. The active sites are always located in *α* subunits and contain the binuclear nickel centre, in which the Ni–Ni distances range from 3.5 to 3.7 Angstrom [4].

Urease as the most characteristic feature of *Helicobacter pylori* constitutes 5–10% of the bacteria’s proteins. *H. pylori* a microaerophilic, gram-negative spiral bacterium which was first detected in 1984 by Marshall et al, is one of the most common chronic bacterial pathogens in humans [5]. Approximately more than 50% of people in the world are infected with it, and its prevalence is significantly higher in developing countries in compare with the developed ones. *H. pylori* infection is an important etiologic impetus usually leading to chronic gastritis, gastro duodenal ulcer and low grade gastric mucosa-associated lymphoid tissue lymphoma. Epidemiological data show that high *H. pylori* infection rate, result in the incidence of gastric cancer and adenocarcinoma [6,7]. Urease catalyzes the hydrolysis of urea to produce ammonia and carbon dioxide, and the most crucial role is to protect the bacteria in the acidic environment of the stomach [8]. It has been also reported that ammonia and monochloramine, which is a reaction product of ammonia and hypochlorous acid, exhibit potent toxicity in gastric epithelium [9]. Moreover, it has been demonstrated that *H. pylori* lacking urease activity are incapable of causing infection in animal models. Thus, it is most likely that urease is essential for bacterial colonization and perhaps the pathogenesis of related disease in vivo.

World Health Organization (WHO) has categorized *H. pylori* as a class 1 carcinogen [10]. Fortunately, its eradication with antibiotics can result in ulcer healing, prevent peptic ulcer recurrence and reduce the prevalence of gastric cancer in high-risk populations. However, it is not always successful because of its resistance to one or more antibiotics and other factors such as poor patient compliance, undesirable side effects of the drugs and significant cost of combination therapy [11]. Wolle et. al. reported that approximately 20% of the patients undergoing antibiotics therapy would experience therapeutic failure [12]. In developing countries, since the application of antibiotics is still under a poor management as a whole, there is a growing need for finding new anti-*H. pylori* agents that can hopefully eradicate the invasion and presence of survived *H. pylori* strains to avoid relapse of gastric ulcer. Hence, a considerable variety of studies involving tests for medicinal plants showing antimicrobial activity and discrepant susceptibility test results are available due to variations in the methods and conditions used for its susceptibility testing.

One of the best sources of new substances to treat *H. pylori* is natural products and their derivatives [13]. Variety of techniques such as synthesizing [6], and also molecular modeling and virtual screening methods [14,15] have been applied to find possible urease inhibitors. The biological activity of plant-derived substances may be considered as a source of new anti-*H. pylori* drugs come from different classes of compounds and are characterized by the diversity of their structures. Therefore, almost all traditional Iranian herbal medicines that are used as remedies and sold as medicines to manage different diseases were screened to discover possible plant-derived urease inhibitors.

## Methods

### Materials

Sodium nitroprusside (sodium pentacyanonitrosyloferrate III) and urease (EC 3.5.1.5) from Jack beans were purchased from Sigma (St. Louis, MO, USA). All other chemicals were of analytical reagent grade from Merck. Deionized water was used in all experiments. Potassium phosphate buffer (100 mM), pH 7.6 was prepared in distilled water.

The studied plants were collected from local medicinal herb shops, Tehran, Iran (June 2010) and were identified by one of our authors of the presented article (F. Mojab). The authenticated samples were deposited in the Herbarium of Shahid Beheshti University of Medical Sciences.

### Extract preparation

10 g of air-dried and powdered plant material was extracted in 10 ml, 50:50 methanol: water at room temperature for 24 hrs. The resulting liquid extract was filtered and concentrated to dryness under reduced pressure. The dry extracts were stored at -20°C till used [16].

### Determination of urease activity

All extracts were tested for urease inhibitory at concentration of 10 mg/ml by the modified spectrophotometric method developed by Berthelot reaction [17]. For herbal extracts that were proven to exert significant inhibition and also for positive controls, inhibitory assays were performed. The plant extracts were tested in a concentration range of 0 to 10 mg/ml. Hydroxyurea was used as standard inhibitor.

The solution assay mixture consisted of urea (30 mM) and (100 μl) crud extract with a total value of 950 μl. The reactions were initiated by the addition of 50 μl of urease enzyme solution in phosphate buffer (100 mM, pH 7.6, 1 mg/ml). Urease activity was determined by measuring ammonia concentration after 15 minutes of enzymatic reaction. The ammonia was determined using 500 μl of solution A (contained 5.0 g phenol and 25 mg of sodium nitroprusside in 500 ml of distilled water) and 500 μl of solution B (contained of 2.5 g sodium hydroxide and 4.2 ml of sodium hypochlorite 5% in 500 ml of distillated water) at 37°C for 30 minutes. The absorbance was read at 625 nm. Activity of uninhibited urease was designated as the control activity of 100%.

### Data processing

The extent of the enzymatic reaction was calculated based on the following equation:

$$I\left(\%\right)=\left[1–\left(T/C\right)\right]*100$$

Where *I* (%) is the inhibition of the enzyme, *T* (test) is the absorbance of the tested sample (plant extract or positive control in the solvent) in the presence of enzyme, *C* (control) is the absorbance of the solvent in the presence of enzyme. Data are expressed as mean ± standard error (SD) and the results were taken from at least three times.

### Determination of IC_50 _values

IC_50 _values (concentration of test compounds that inhibits the hydrolysis of substrates by 50%) were determined by studying the extracts urease inhibitory activity at their different concentrations in comparison to their individual positive control employing spectrophotometric measurement. IC_50 _values were obtained from dose-response curves by linear regression, using Graphpad software, prism 5.

## Results and discussion

Medicinal plants as an appropriate and renewable source of active chemical compounds can be used as templates to discover new lead compounds. Doxorubicin, vincristine, and taxol, are examples of these herbal compounds which are clinically applied. According to the literature, 50% of commercially presented medicines in 1985 was from herbal origins [18]. Gastrointestinal diseases, especially gastric, duodenal and peptic ulcer, arise from different factors, in particular microbial agent *H. pylori*. Common multi-drug therapies not only have side effects, but are also expensive. On the other hand, the probability of drug resistance occurrence and disease retrogression is quite concerning. Already reported studies have shown that herbal compounds have the ability to prevail this microbe. Among the studied herbal essences and extracts, many did not exceed the study level due to production limits, toxicity and impossibility of drug form preparation. Majority of the researches have focused on ways to inhibit the bacteria growth or it’s elimination from the culture, while a few has particularly concerned inhibition of urease enzyme which is responsible for the bacteria defense system against the stomach very acidic medium.

Specific inhibition or reduction of urease enzyme activity would result in an increased sensitivity of the bacteria in acidic medium and therefore it’s natural elimination by stomach acidic condition or the body immune system.

In the presented study, urease enzyme inhibition potency of 137 herbal extracts was investigated from which 37 extracts have shown inhibitory activity up to more than 70% in the concentration of 10 mg/ml (Table 1). Further examinations and IC_50 _determination revealed that *Sambucus ebulus, Rheum ribes, Pistachia lentiscus*, *Myrtus communis*, *Myristica fragrans*, *Areca catechu*, *Cinnamomum zeylanicum*, *Citrus aurantifolia* and *Nicotiana tabacum* extracts inhibit urease enzyme in concentrations less than 500 μg/ml. It should also be mentioned that *C. zeylanicum*, *M. chamomilla and M. spicata* are already used as gastrointestinal remedies and this research has proved that these herbs can inhibit urease activity and prevent gastric upsets. Names of the studied plants and the 37 final more active extracts are presented in Table 2. As it is shown, the most potent urease inhibitory was observed for *S. ebulus* and *R. ribes* with IC_50 _values less than 100 μg/ml.

**Table 1:** Urease inhibitory activity of plants extract at concentration of 10 mg/ml

	**Scientific name**	**Plant family**	**Common name in English**	**Common name in Persian**	**Part used in traditional**	**Inhibition (%)**
1.	*Abrus precatorius*	Fabaceae	Paternoster Seed	Cheshm-e khorus	Seed	9.21 ± 0.04
2.	*Acacia Senegal*	Fabaceae	Gum Arabic	Samgh-e arabi	Gum	12.81± 0.09
3.	*Acanthophyllum squarrosum*	Asparagaceae	Soap Root	Chubak	Root	14.15 ± 0.02
4.	*Alpinia officinarum*	Zingiberaceae	Galangal	Khulanjan	Rhizome	41.75 ± 0.05
5.	*Althaea officinalis*	Malvaceae	Hollyhoch	Khatmi	Flower	20.94 ± 0.06
6.	*Alyssum homolocarpum*	Brassicaceae	Madword & Pepper Weed	Qodume	Seed	13.57± 0.12
7.	*Amaranthus lividus*	Amaranthaceae	Cock's Comb Seed	Tokhm-e tajkhorus	Seed	17.48 ± 0.11
8.	*Anethum graveolins*	Apiaceae	Dill Seed	Tokhm-e shevid	Seed	37.50 ± 0.03
9.	*Apium graveolens*	Apiaceae	Celery Seed	Tokhm-e karafs	Seed	2.43 ± 0.01
10.	*Aquilaria sinensis*	Thymelaeaceae	Agarwood	Udeqamari	Fruit	32.03 ± 0.08
11.	*Arctium Lappa*	Asteraceae	Burdock Root	Bâbââdam	Root	19.99 ± 0.08
12.	*Areca catechu*	Arecaceae	Betel Nuts	Fufel	Fruit	96.67 ± 0.01
13.	*Artemisia absinthium*	Asteraceae	Worm Wood	Afsantin	Herb	52.50 ± 0.06
14.	*Artemisia dracunculus*	Asteraceae	Tarragon	Tarkhon	Leaf	57.53 ± 0.03
15.	*Asperugo procumbens*	Boraginaceae	German Madwort	Bâdranjbuye	Herb	12.43 ± 0.02
16.	*Astragalus arbusculinus*	Fabaceae	Sarcocola	Anzarut	Gum	17.68 ± 0.06
17.	*Astragalus gossypinus*	Fabaceae	Gum Tragacanth	Katirâ	Gum	1.33 ± 0.02
18.	*Bambusa vulgaris*	Poaceae	Golden Bamboo	Tabâshir sadaf	Secretions	12.81 ± 0.04
19.	*Brassica nigra*	Brassicaceae	Mustard	Khardel	Seed	27.63 ± 0.01
20.	*Calendula officinalis*	Asteraceae	Marigold	Hamishe bahar	Flower	0.16 ± 0.06
21.	*Calendula* sp*.*	Asteraceae	Marigold	Hamishe bahar	Flower	8.21 ± 0.07
22.	*Camellia sinensis*	Theaceae	Green Tea	Chây-e sabz	Leaf	89.40 ± 0.02
23.	*Camellia sinensis*	Theaceae	Green Tea	Châyeparsefid	Twig	90.45 ± 0.01
24.	*Cannabis sativa*	Cannabaceae	Hemp Seed	Shâhdane	Seed	9.71 ± 0.02
25.	*Capsicum annuum*	Solanaceae	Red Pepper	Felfel-e qermez	Fruit	99.01 ± 0.01
26.	*Carthamus tinctorius*	Asteraceae	Saf	Golrang	Flower	50.78 ± 0.04
27.	*Cassia angustifolia*	Fabaceae	Senna	Sena	Leaf	3.29 ± 0.03
28.	*Celosia cristata*	Amaranthaceae	Cockscomb	Gol-e halva	Flower	82.55 ± 0.03
29.	*Centaurea* sp*.*	Asteraceae	Centaurea	Gol-e gandom	Flower	70.33 ± 0.02
30.	*Chenopodium botrys*	Amaranthaceae	Lamb's Quarter	Dermane-e torki	Herb	15.13 ± 0.04
31.	*Cichorium intybus*	Asteraceae	Chicory	Kâsni	Herb	40.55 ± 0.04
32.	*Cinchona officinalis*	Rubiaceae	Cinchona	Gne gne	Bark	67.03 ± 0.02
33.	*Cinnamomum camphora*	Lauraceae	Camphre	Kâfur	Camphor	10.14 ± 0.08
34.	*Cinnamomum cassia*	Lauraceae	Cassia	Salikhe	Bark	91.19 ± 0.02
35.	*Cinnamomum zeylanicum*	Lauraceae	Cinnamon	Darchin	Bark	84.22 ± 0.05
36.	*Citrus aurantifolia*	Rutaceae	Limu Fruit	Limu ammâni	Fruit	99.02 ± 0.02
37.	*Citrus aurantium*	Rutaceae	Bitter Orange Peel	Khalâl-e nârenj	Rind	1.43 ± 0.05
38.	*Citrus bigardia*	Rutaceae	Orange	Gol-e nârenj	Twig	24.31 ± 0.03
39.	*Colchicum macrophyllum*	Colchicaceae	Colchicum Corms	Suranjan	Corm	9.44 ± 0.08
40.	*Commiphora molmol*	Burseraceae	Myrrh	Morr-e Makki	Gum	8.22 ± 0.04
41.	*Crataegus microphylla*	Rosaceae	Hawthorn	Sorkhe valik	Flower	82.19 ± 0.03
42.	*Curcuma zedoaria*	Zingiberaceae	Zedoary	Zorombad	Seed	4.70 ± 0.06
43.	*Cuscuta epithymum*	Convolvulaceae	Hellweed	Aftimun	Herb	9.66 ± 0.01
44.	*Cymbopogon*	Poaceae	Lemongrass	Putar	Root	14.02 ± 0.03
45.	*Descureania*	Brassicaceae	Flixweed Seed	Khakshir	Seed	21.81 ± 0.01
46.	*Diplotaenia damavendica*	Apiaceae	Diplotaenia	Gozal	Seed	12.59 ± 0.06
47.	*Doronicum bracteatum*	Asteraceae	Doronicum	Darunj-e aqrabi	Herb	10.73 ± 0.01
48.	*Dracaena cinnabari*	Asparagaceae	Dragon Blood	Khone siyavosh	Gum	49.49 ± 0.13
49.	*Dracocephalum*	Lamiaceae	Moldavian Balm	Badrashbi	Twig	3.95 ± 0.01
50.	*Echinophora platyloba*	Apiaceae	Echinophora	Khosharize	Herb	17.48 ± 0.01
51.	*Echium amoenum*	Boraginaceae	Ox tongue Flower	Gol-e gâvzabân	Flower	31.66 ± 0.02
52.	*Elaeagnus angustifolia*	Elaeagnaceae	Oleaster	Senjed	Fruit	4.67 ± 0.14
53.	*Elaeagnus angustifolia*	Elaeagnaceae	Oleaster	Gol-e senjed	Flower	27.45 ± 0.01
54.	*Elettaria cardamomum*	Zingiberaceae	Cardamon	Hel sabz	Fruit	13.16 ± 0.04
55.	*Elletaria cardamomum*	Zingiberaceae	Cardamon	Hel sefid	Fruit	6.80 ± 0.07
56.	*Elletaria cardamomum*	Zingiberaceae	Cardamon	Hel siyah	Fruit	5.78 ± 0.071
57.	*Equisetum arvense*	Equisetaceae	Horse Tail	Dom-e asb	Stem	52.35 ± 0.05
58.	*Eruca sativa*	Brassicaceae	Rocket	Tokhm-e mandâb	Seed	13.28 ± 0.05
59.	*Eucalyptus* sp.	Myrtaceae	Eucalyptus	Okaliptus	Leaf	47.92 ± 0.01
60.	*Euphorbia* sp.	Euphorbiaceae	Euphorbia	Gav kosh	Herb	68.94 ± 0.03
61.	*Ferula assa-foetida*	Umbelliferae	Assa-Foetid	Anqoze	Gum	34.07 ± 0.04
62.	*Helicteres isora*	Malvaceae	Screw Tree Pod	Bahmanpich	Fruit	8.18 ± 0.02
63.	*Heracleum persicum*	Apiaceae	Cow Parsnip Friut	Golpar	Fruit	10.27 ± 0.02
64.	*Hibiscus gossypifolius*	Malvaceae	Rose Mallow	Chay-e Makki	Herb	96.28 ± 0.02
65.	*Humulus lupulus*	Cannabaceae	Hops	Râzak	Twig	54.85 ± 0.02
66.	*Hypericum perforatum*	Hypericaceae	St.John's Wort	Alaf-e chay	Herb	97.99 ± 0.02
67.	*Juglans regia*	Juglandaceae	Walnut Shell	Pust-e vasat-e gerdo	Septum	93.62 ± 0.01
68.	*Juglans regia*	Juglandaceae	Walnut Shell	Pust-e gerdo	Rind	1.27 ± 0.06
69.	*Juniperus Sabina*	Cupressaceae	Sabine	Abhal	Fruit	19.63 ± 0.01
70.	*Lactuca sativa*	Asteraceae	Lettuce	Tokhm-e Kâhu	Seed	2.93 ± 0.04
71.	*Lawsonia inermis*	Lythraceae	Henna	Hana	Leaf	54.00 ± 0.06
72.	*Levisticum officinalis*	Apiaceae	Lovage	Anjadân romi	Seed	10.00 ± 0.06
73.	*Linum usitatissimum*	Linaceae	Lineseed	Tokhm-e katan	Seed	2.71 ± 0.18
74.	*Malabaila secacule*	Apiaceae	Parsnip	Dogho	Root	18.18 ± 0.04
75.	*Malva sylvestris*	Malvaceae	Common Mallow	Gol-e panirak	Flower	14.15 ± 0.05
76.	*Matricaria chamomilla*	Asteraceae	Chamomile	Bâbon-e shirazi	Herb	87.21 ± 0.01
77.	*Melissa officinalis*	Lamiaceae	Balm	Barangbu	Herb	46.22 ± 0.05
78.	*Mentha spicata*	Lamiaceae	Mint	NaAna	Leaf	93.89 ± 0.01
79.	*Myristica fragrans*	Myristicaceae	Nutmeg	Joz-e buya	Fruit	78.19 ± 0.01
80.	*Myrtus communis*	Myrtaceae	Myrtle	Murd	Leaf	72.99 ± 0.01
81.	*Nasturdium officinalis*	Brassicaceae	Watercress	Boolâgoti	Leaf	74.00 ± 0.03
82.	*Nerium Oleander*	Apocynaceae	Nerium	Gol-e kharzahre	Flower	84.62 ± 0.01
83.	*Nicotiana Tabacum*	Solanaceae	Tobacco	Tutun	Leaf	52.77 ± 0.03
84.	*Nicotiana tabacum*	Solanaceae	Tobacco	Tutun	Stem	75.26 ± 0.05
85.	*Nymphaea alba*	Nymphaeaceae	White Lotus	Gol-e nilofar	Flower	97.86 ± 0.01
86.	*Ocimum basilicum*	Lamiaceae	Basil	Reyhan-e banafsh	Leaf	19.61 ± 0.05
87.	*Ocimum basilicum*	Lamiaceae	Basil	Reyhan-e sabz	Leaf	0.41 ± 0.01
88.	*Oenothera biennis*	Onagraceae	Evening Star	Gol-e maghrebi	Flower	3.95 ± 0.04
89.	*Olea europea*	Oleaceae	Olive Leaf	Barg-e zyton	Leaf	72.30 ± 0.01
90.	*Orchis latifolia*	Orchidaceae	Oriental Salp	SaAlab-e panjei	Root	18.90 ± 0.02
91.	*Orchis mascula*	Orchidaceae	Male Orchis	SaAlab-e qolvei	Root	3.16 ± 0.04
92.	*Papaver Rhoeas*	Papaveraceae	Corn Poppy	Pust-e shaghayegh	Rind	27.25 ± 0.12
93.	*Papaver Rhoeas*	Papaveraceae	Corn Poppy	Gol-e shaghayegh	Flower	97.50 ± 0.01
94.	*Papaver somniferum*	Papaveraceae	Opium Poppy	Khashkhash	Seed	4.79 ± 0.03
95.	*Papaver somniferum*	Papaveraceae	Opium Poppy	Khashkhash	Fruit	35.95 ± 0.02
96.	*Passiflora caerulea*	Passifloraceae	Passion Flower	Gol-e sâAty	Flower	46.90 ± 0.008
97.	*Pelargonium graveolens*	Geraniaceae	Geranium	Barg-e atr	Leaf	92.19 ± 0.01
98.	*Pelargonium graveolens*	Geraniaceae	Rose Pelargonium	Gol-e atr	Flower	96.87 ± 0.02
99.	*Pterocarpus rubra*	Fabaceae	Mukwa	Sandal-e sorkh	Bark	91.75 ± 0.01
100.	*Petroselinum hortense*	Apiaceae	Parsley Seed	Tokhm-e jafari	Seed	50.35 ± 0.03
101.	*Pistachio lentiscus*	Anacardiaceae	Lentisk Pistache	Mastaki	Gum	92.37 ± 0.01
102.	*Pistacia vera*	Anacardiaceae	Pistachio Nut Shell	Pust-e peste	Rind	97.71 ± 0.01
103.	*Plantago major*	Plantaginaceae	Great Plantain	Bârhang	Seed	4.69 ± 0.11
104.	*Polyporus officinalis*	Fomitopsidaceae	White Agaric	Ghariqun	Fungi	19.97 ± 0.06
105.	*Portulaca oleracea*	Portulacaceae	Common Purslane Seed	Tokhm-e khorfe	Seed	7.19 ± 0.03
106.	*Prunus persica*	Rosaceae	Peach	Barge-e holo	Fruit	9.47 ± 0.11
107.	*Punica granatum*	Lythraceae	Pomegranate Flower	Golnar	Flower	99.90 ± 0.01
108.	*Punica granatum*	Lythraceae	Pomegranate	Golnar	Rind	99.90 ± 0.01
109.	*Quercus infectoria*	Fagaceae	Oak Gall	Qolqaf	Gall	53.97 ± 0.02
110.	*Quercus infectoria*	Fagaceae	Oak Fruit Hull	Jaft	Rind	98.84 ± 0.02
111.	*Rheum ribes*	Polygonaceae	Rhubarb	Rivâs	Root	98.93 ± 0.01
112.	*Rosa centifolia*	Rosaceae	Damask Rose	Gol-e sorkh	Flower	97.51 ± 0.01
113.	*Rosa foetida*	Rosaceae	Rosa Lutea	Gol-e zard	Flower	89.19 ± 0.023
114.	*Rosmarinus angustifolia*	Lamiaceae	Pine Rosemary	Rozmary-e aklilaljabal	Leaf	22.51 ± 0.02
115.	*Rubia tinctorium*	Rubiaceae	Madder Root	Ronas	Root	37.31 ± 0.02
116.	*Ruta graveolens*	Rutaceae	Rue	Sodab	Leaf	27.91 ± 0.04
117.	*Saccharum officinarum*	Poaceae	Sugar Cane	Shekar-e sorkh	Mann	35.04 ± 0.06
118.	*Salix aegyptiaca*	Salicaceae	Aegyption Willow	Bidmeshk	Flower	17.05 ± 0.02
119.	*Salix* sp*.*	Salicaceae	Whitewillow	Pust-e bid	Bark	38.10 ± 0.01
120.	*Salvia hydrangea*	Lamiaceae	Mountain Sage	Gol-e arune	Flower	91.09 ± 0.01
121.	*Salvia macrosiphon*	Lamiaceae	Willd Sage Seeds	Thokhm-e marv	Seed	2.86 ± 0.04
122.	*Sambucus ebulus*	Adoxaceae	Dwarf Elder	Tarâsit	Fruit	99.70 ± 0.01
123.	*Santalum album*	Santalaceae	Sandalwood	Sandal-e sefid	Bark	58.69 ± 0.02
124.	*Satureja hortensis*	Lamiaceae	Savory Seed	Tokhm-e marze	Seed	35.77 ± 0.04
125.	*Scrophularia striata*	Scrophulariaceae	Striata Figwort	Mokhallace	Stem& Flower	16.47 ± 0.05
126.	*Sinapis alba*	Brassicaceae	White Mustard	Khardal-e sefid	Seed	39.77 ± 0.06
127.	*Spinacia oleracea*.	Amaranthaceae	Spinach Seed	Tokhm-e esfenaj	Seed	19.76 ± 0.04
128.	*Taraxacum* sp*.*	Asteraceae	Dandelion	Ghasedak	Flower	14.83 ± 0.01
129.	*Thymus kotschyanus*	Lamiaceae	Kotschyam Thyme	Avishan	Herb	17.94 ± 0.01
130.	*Tilia platyphyllos*	Malvaceae	Linden	Zirfun	Leaf & Flower	25.79 ± 0.01
131	*Trigonella foenum-graecum*	Fabaceae	Fenugreek Seed	Tokhm-e shanbalile	Seed	44.02 ± 0.02
132.	*Triticum sativum*	Poaceae	Wheat	Sabos-e ghandom	Husk	16.14 ± 0.04
133.	*Tussilago farfara*	Asteraceae	Colt′s-foot	Pakhari	Herb	69.08 ± 0.01
134.	*Veratrum album*	Melanthiaceae	White Hellebore	Kharbogh	Leaf	96.85 ± 0.06
135.	*Verbascum georgicum*	Scrophulariaceae	Mullein	Dom-e gav	Leaf	30.40 ± 0.03
336.	*Verbascum* sp.	Scrophulariaceae	Mullein	Marg-e mâhi	Fruit	0.82 ± 0.05
137.	*Ziziphus vulgaris*	Rhamnaceae	Jujube	Annâb	Fruit	26.34 ± 0.01
138.	Hydroxyurea	-------------	------------	----------	Reference compound	100 ± 0.01

**Table 2:** **IC**_**50 **_**and medicinal uses of most active plants**

	**Scientific name**	**Effects & medicinal uses**	**IC**_**50 **_**(μg/ml)**	**Std. Error log IC**_**50**_
1.	*A. catechu*	Anthelmintic, gastric tonic	216	0.01
2.	*C. cristata*	Styptic, depurative, sedative, constipating, antibacterial, febrifuge,	6175	0.68
3.	*C. annuum*	Anti flatulence, gout, gastric tonic, paralysis	751	0.14
4.	*C. aurantifolia*	Appetitive, anti-flatulence, analgesic	432	0.06
5.	*C. cassia*	Gastric tonic, anti-spasmodic, anti-flatulence	867	0.05
6.	*C. microphylla*	Anti-flatulence, gastric tonic	665	0.14
7.	*C. sinensis*	Anti-bacterial, anti diarrhea, diuretic, astringent, reduce cholesterol	579	0.04
8.	*C. zeylanicum*	Gastric tonic, anti-flatulence, ‘anti-spasmodic	361	0.02
9.	*C. sinensis*	Anti-diarrhea, diuretic, astringent, anti-bacterial, reduce cholesterol	1314	0.04
10.	*Cetaurea* sp*.*	Anti-inflammatory, astringent, emmenagogue, sedative	5152	0.05
11.	*H. gossypifolius*	Analgesic, anti-tussive, demulcent, diuretic, febrifuge, highly emollient, slightly laxative and odontalgic, anti-inflammations and laryngitis,	819	0.01
12.	*H. perforatum*	Astringent, analgesic, anti-inflammator, anti-anxiety aphrodisiac	3509	0.10
13.	*J. regia*	Anti-inflammatory, astringent, anti-spasmodic	1271	0.08
14.	*M. chamomilla*	Anti-inflammation, appetitive, and aids digestion and sleep, acts as a diuretic and nerve tonic.	3188	0.02
15.	*M. fragrans*	Anti-flatulence appetitive ‘anti-spasmodic’ antiseptic, analgesic, anti-inflammatory	215	0.15
16.	*M. spicata*	Analgesic, Anti-spasmodic, anti-flatulence	7822	0.17
17.	*M. communis*	Antiseptic, disinfectant, expectorant, deodorizer	170	0.04
18.	*N. officinale*	Diuretic, expectorant, purgative, hypoglycemic, odontalgic, stimulant, tonic and stomachic	2055	0.19
19.	*N. alba*	Astringent, antiseptic, anesthetic, aphrodisiac, sedative, used for gastrointestinal disorders and jaundice	820	0.19
20.	*N. Oleander*	Dermatitis, abscesses, eczema, psoriasis, sores, warts, corns, ringworm, scabies, herpes, skin cancer, asthma, dysmenorrheal, epilepsy, malaria,	9877	0.26
21.	*N. tabacum*	Anti-spasmodic, diuretic, sedative, sialagogue	473	0.15
22.	*O. europea*	Hypotensive, diuretic, hypoglycemic	2857	0.06
23.	*P. granatum*(Rind)	Hypoglycemic, anti-cancer, anthelmintic	1484	0.10
24.	*P. granatum* (Flower)	Hypoglycemic, anti-cancer, anthelmintic	1331	0.11
25.	*P. graveolens*	Anti-inflammatory, antiseptic, aromatherapy, astringent, anti- cancer, sedative	976	0.03
26.	*P. graveolens*	Analgesic, anti-Bacterial, anti-Depressant, anti-inflammatory, antiseptic, astringent, diuretic, insect repellent, refreshing, relaxing, sedative, styptic, tonic	1242	0.14
27.	*P. lentiscus*	Antibacterial	121	0.03
28.	*P. Rhoeas*	Anodyne, emmenagogue, emollient, expectorant, hypnotic, sedative, tonic	5636	0.04
29.	*P. rubra*	Astringent, tonic	930	0.06
30.	*P. vera*	Aphrodisiac, anti-anxiety	4687	0.12
31.	*Q. infectoria*	Gingivitis, infectoria, anti-diabetic, anti-tremorine, local anesthetic, antiviral, antibacterial, antifungal.	1214	0.12
32.	*R. centifolia*	Anti-inflammatory, antispasmodic, aphrodisiac, astringent, depurative, laxative, analgesic, appetitive	544	0.07
33.	*R. foetida*	Heart diseases, digestive, skin diseases, muscular pains, anti-parasite	2441	0.19
34.	*R. ribes*	Appetitive, astringent, anti bacteria, anti depressive and used to treat diabetes, hemorrhoids, ulcer, diarrhea	92	0.06
35.	*S. hydrangea*	anti-flatulence, astringent, anti-spasmodic	2960	0.11
36.	*S. ebulus*	Anti-inflammatory, antinociceptive, anti-cancer, anti-angiogenic, anti-oxidative	57	0.05
37.	*V. album*	Analgesic, anthelmintic, cathartic, emetic, expectorant, hypnotic	1037	0.07
38.	hydroxyurea		37	0.02

**Figure 1:**
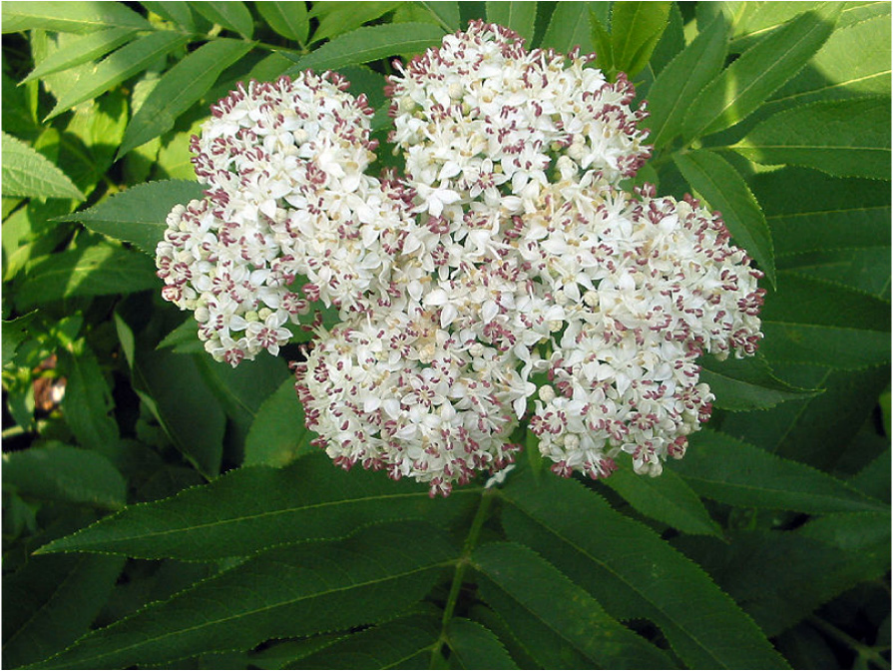
***Sambucus ebulus *in flowering stage **[19]**.**

*S. ebulus* (Figure 1) is a native perennial herb of the Adoxaceae family [19]. It has been prescribed in traditional medicines for the treatment of inflammatory reactions, such as hemorrhoid, bites and sore-throat. In addition, *S. ebulus* has been shown to have anti-inflammatory, antinociceptive, anti-cancer, anti-angiogenic and anti-oxidative activities. Ebulitin, ebulin 1, flavonoid, anthocyanin and other components have been isolated from *S. ebulus* and identified as active ingredients of biological and pharmacological activities [20]. The anti-*H. pylori* effect of the *S. ebulus* extract was observed by using the agar dilution method [13].

**Figure 2:**
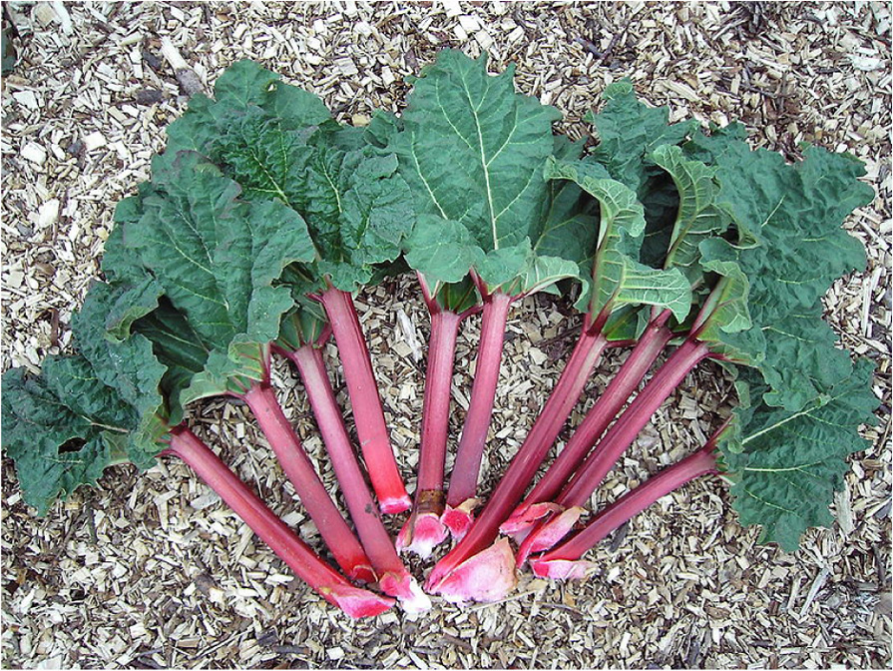
***Rheum ribes *leaf **[21]**.**

*R. ribes* (Figure 2) is a hardy perennial, cultivated in some temperate countries for its edible red leaf stalks [21]. It is used to treat diabetes, hemorrhoids, ulcer, diarrhea, and expectorant activity reported. The efficacy and safety of a hydroalcohlic extract of *R. ribes* in treatment of mild to moderate major depression disorder has been investigated and the observations show some anti depressive effects. The methanolic extract of *R. ribes* have demonstrated anti-ulcer activity comparable with standard drugs cimetidine [22].

According to strong inhibitory activity of the herbs presented in Table 2, simultaneous application of theses herbs and the medicines prescribed in gastrointestinal disease therapies would fasten the treatment. Additionally, isolation of active compounds and further investigation of each isolated compound against urease activity would lead to new chemical structures which may have the potency to inhibit urease activity.

## Competing interests

There are no other conflicts of interest related to this publication.

## Authors’ contributions

All authors contributed to the concept and design, making and analysis of data, drafting, revising and final approval. MA and BY are responsible for the study registration. FM is responsible for plants identification and collection. FN and KB carried out plant extraction and enzymatic tests and drafted manuscript. MHR, FN, MA and BY participated in collection and/or assembly of data, data analysis, interpretation and manuscript writing. All authors read and approved the final manuscript.

## References

[B1] Jabri E Lee MH Hausinger RP Karplus PA Preliminary crystallographic studies of urease from jack bean and from Klebsiella aerogenes J Mol Biol 1992 227 934 937 10.1016/0022-2836(92)90232-9 1404395

[B2] Todd MJ Hausinger RP Purification and characterization of the nickel-containing multicomponent urease from Klebsiella aerogenes J Biol Chem 1987 262 5963 5967 3553184

[B3] Koper TE El-Sheikh AF Norton JM Klotz MG Urease-encoding genes in ammonia-oxidizing bacteria Appl Environ Microbiol 2004 70 2342 2348 10.1128/AEM.70.4.2342-2348.2004 15066830 PMC383159

[B4] Krajewska B Ureases I Functional, catalytic and kinetic properties: A review J Mol Catal B: Enzym 2009 59 9 21 10.1016/j.molcatb.2009.01.003

[B5] Owen RJ Bacteriology of *Helicobacter pylori* Baillieres Clin Gastroenterol 1995 9 415 446 10.1016/0950-3528(95)90041-1 8563046

[B6] Kosikowska P Berlicki L Urease inhibitors as potential drugs for gastric and urinary tract infections: a patent review Expert Opin Ther Pat 2011 21 945 957 10.1517/13543776.2011.574615 21457123

[B7] Mobley HL Hu LT Foxal PA *Helicobacter pylori* urease: properties and role in pathogenesis Scand J Gastroenterol Suppl 1991 187 39 46 1775923

[B8] Stingl K Altendorf K Bakker EP Acid survival of *Helicobacter pylori*: how does urease activity trigger cytoplasmic pH homeostasis? Trends Microbiol 2002 10 70 74 10.1016/S0966-842X(01)02287-9 11827807

[B9] Dekigai H Murakami M Kita T Mechanism of *Helicobacter pylori*-associated gastric mucosal injury Dig Dis Sci 1995 40 1332 1339 10.1007/BF02065547 7781456

[B10] Forman D *Helicobacter pylori* and gastric cancer Scand J Gastroenterol Suppl 1996 215 48 51 8722382

[B11] O'Connor A Gisbert JP McNamara D O'Morain C Treatment of *Helicobacter pylori* infection Helicobacter 2011 1 16 Suppl 53 58 21896086 10.1111/j.1523-5378.2011.00881.x

[B12] Wolle K Malfertheiner P Treatment of *Helicobacter pylori* Best Pract Res Clin Gastroenterol 2007 21 315 324 10.1016/j.bpg.2006.11.001 17382279

[B13] Yesilada E Gurbuz I Shibata H Screening of Turkish anti-ulcerogenic folk remedies for anti-*Helicobacter pylori* activity J Ethnopharmacol 1999 66 289 293 10.1016/S0378-8741(98)00219-0 10473175

[B14] Azizian H Nabati F Sharifi A Siavoshi F Mahdavi M Amanlou M Large-scale virtual screening for the identification of new *Helicobacter pylori* urease inhibitor scaffolds J Mol Model 2011 3 1 11 10.1007/s00894-011-1310-2 22139480

[B15] Azizian H Bahrami H Pasalar P Amanlou M Molecular modeling of *Helicobacter pylori* arginase and the inhibitor coordination interactions J Mol Graph Model 2010 28 626 635 10.1016/j.jmgm.2009.12.007 20080052

[B16] Gennaro AR Remington’s Pharmaceutical Sciences 1985 Pennsylvania: Mack Publishing Company 1516

[B17] Nabati F Habibi-Rezaei M Amanlou M Moosavi-Movahedi AA Dioxane enhanced immobilization of urease on alkyl modified nano-porous silica using reversible denaturation approach J Mol Catal B: Enzym 2011 70 17 22 10.1016/j.molcatb.2011.01.014

[B18] Lipp FJ The efficacy, history, and politics of medicinal plants Altern Ther Health Med 1996 2 36 41 8795920

[B19] Wikipedia contributors "Danewort" Wikipedia, The Free Encyclopedia http://en.wikipedia.org/wiki/Danewort, (accessed September 25, 2012)

[B20] Shokrzadeh M Saravi S The chemistry, pharmacology and clinical properties of *Sambucus ebulus*: a review J Med Plants Res 2010 4 95 103

[B21] Wikipedia contributors "Rheum" Wikipedia, The Free Encyclopedia http://en.wikipedia.org/wiki/Rheum_(plant), (accessed September 25, 2012)

[B22] Sindhu RK Kumar P Kumar J Kumar A Arora S Investigations into the anti-ulcer activity of *Rheum ribes* linn leaves extracts Int J Pharm Pharm Sci 2010 2 90 92

